# Quality Comparative Evaluation of Eungyosan Formulations by a Validated HPLC–PDA Method for 11 Marker Components

**DOI:** 10.3390/molecules31121991

**Published:** 2026-06-07

**Authors:** Chang-Seob Seo

**Affiliations:** KM Science Research Division, Korea Institute of Oriental Medicine, Daejeon 34054, Republic of Korea; csseo0914@kiom.re.kr; Tel.: +82-42-868-9361

**Keywords:** Eungyosan, HPLC–PDA, quality evaluation, herbal formulation

## Abstract

Eungyosan (EGS) is a traditional multi-herbal formulation widely used for the treatment of respiratory diseases; however, its quality control remains challenging due to its complex chemical composition. This study aimed to develop and validate a high-performance liquid chromatography coupled with photodiode array detection (HPLC–PDA) method for simultaneous determination of 11 representative marker compounds in EGS and to apply the method to the comparative quality evaluation of laboratory-prepared and commercial EGS formulations. Chromatographic conditions were optimized, and the marker compounds were selected based on their herbal origin, phytochemical relevance, and chromatographic detectability. The method was validated in terms of linearity, sensitivity (limits of detection and quantification), precision, accuracy, and stability. All analytes exhibited excellent linearity (coefficient of determination > 0.9999), along with satisfactory precision (relative standard deviation < 2%) and recovery (95.64–105.69%). The validated method was successfully applied to a laboratory-prepared extract and three commercial granule formulations. Considerable differences in the levels of marker compounds were observed among the samples; several marker compounds were either not detected or could not be quantified because of UV spectral mismatch in certain commercial products. These findings demonstrate variability in the chemical composition of the tested EGS formulations and highlight the usefulness of the validated HPLC–PDA method for comparative quality evaluation of multi-herbal formulations.

## 1. Introduction

Eungyosan (EGS; Yinqiaosan in Chinese) is a traditional multi-herbal formulation composed of several medicinal herbs and has been widely used for the management of respiratory conditions, including the common cold and influenza [1,2]. Owing to its long history of clinical use, EGS is currently manufactured and distributed in various dosage forms, particularly as granule formulations available in the pharmaceutical market [1,3]. In recent years, herbal formulations such as EGS have gained increasing attention during viral outbreaks, including the COVID-19 pandemic [4]. Several studies have reported antiviral and immunomodulatory activities of EGS against influenza viruses [5]. In addition, systems pharmacology studies have explored its potential applications in the context of COVID-19, further highlighting its contemporary importance [6]. Moreover, recent studies have emphasized the importance of standardized quality control strategies for multi-herbal formulations to ensure product consistency, safety, and therapeutic reliability, particularly in the context of increased global interest in herbal medicines during the COVID-19 pandemic [7,8,9].

However, the chemical complexity of multi-herbal formulations presents significant challenges for quality evaluation because the chemical composition may vary depending on raw materials, extraction conditions, and manufacturing processes [10,11]. Previous studies have demonstrated that the chemical composition of EGS can be influenced by extraction methods, highlighting the importance of controlling preparation conditions [12]. Moreover, several constituent compounds of EGS have been associated with various biological activities, supporting their role as important chemical markers for quality evaluation. Therefore, comprehensive and reliable analytical approaches are required to enable systematic characterization and comparative quality evaluation of such complex herbal formulations.

To ensure the quality and consistency of herbal medicines, reliable analytical methods for the simultaneous determination of multiple marker compounds are essential for effective quality control [13,14,15]. High-performance liquid chromatography coupled with photodiode array detection (HPLC–PDA) is widely employed for routine quality control due to its robustness, accessibility, and cost-effectiveness [14]. Although several studies have reported chromatographic analysis or fingerprint-based quality evaluation of herbal formulations, validated methods specifically optimized for the simultaneous determination of multiple representative marker compounds in EGS remain limited, particularly for comparative quality evaluation of commercial formulations [1].

In addition, although EGS is widely available in standardized dosage forms, the chemical characteristics of commercially available EGS products have not been systematically compared. Variations in formulation composition, raw materials, and manufacturing processes may result in differences in the levels of marker compounds among products, potentially affecting quality consistency. Therefore, analytical approaches that enable both reliable quantification and comparative evaluation of multiple products are required.

In this study, an HPLC–PDA method was developed and validated for the simultaneous determination of 11 marker compounds in EGS. The validated method was applied to both a laboratory-prepared extract and commercially available granule formulations for comparative evaluation of their chemical profiles. This study provides a validated and practical analytical approach for the comparative quality evaluation of EGS formulations and contributes to improved quality control of complex multi-herbal products.

## 2. Results

### 2.1. Selection of 11 Marker Compounds

To select appropriate marker compounds for the quality control of EGS using HPLC–PDA, the constituent herbal materials and their representative chemical components were first investigated. Based on literature reports and the known phytochemical profiles of each herb, a total of 24 candidate compounds were initially selected from the major constituent herbs of EGS, including Forsythiae Fructus (*Forsythia viridissima* Lindl.), Lonicerae Flos (*Lonicera japonica* Thunb.), Platycodonis Radix (*Platycodon grandiflorum* A.DC.), Menthae Herba (*Mentha arvensis* L.), Lophatheri Herba (*Lophatherum gracile* Brongn.), Glycyrrhizae Radix et Rhizoma Preparata cum Melle (*Glycyrrhiza uralensis* Fisch.), Schizonepetae Spica (*Schizonepeta tenuifolia* Briq.), Glycine Semen Preparata (*Glycine max* (L.) Merr.), and Arctii Fructus (*Arctium lappa* L.) [16,17,18,19,20,21,22,23,24]. The presence of these candidate compounds was examined under HPLC–PDA conditions using a SunFire C_18_ column (250 × 4.6 mm, 5 μm; Waters, Milford, MA, USA) with a gradient elution system consisting of 0.1% (*v*/*v*) formic acid in water and 0.1% (*v*/*v*) formic acid in acetonitrile. As shown in Figure S1, individual herbal extracts confirmed the detectability of the candidate compounds. Subsequently, these compounds were analyzed in the EGS sample (Figure S2). Among the 24 candidates, 11 compounds—chlorogenic acid, 4-hydroxycinnamic acid, liquiritin apioside, liquiritin, genistin, isochlorogenic acid A, arctiin, matairesinol, arctigenin, glycyrrhizin, and pulegone—were consistently detected with stable and well-resolved peaks. Therefore, these compounds were selected as marker compounds for further analysis. Several selected marker compounds, including chlorogenic acid, liquiritin, arctiin, arctigenin, and glycyrrhizin, have been reported to possess anti-inflammatory, antiviral, immunomodulatory, and respiratory protective activities, which are considered relevant to the therapeutic applications of Eungyosan formulations [25,26,27,28]. Detailed information on the candidate compounds investigated and the rationale for their selection or exclusion is provided in Table S1.

### 2.2. Optimization of Chromatographic Conditions

The chromatographic conditions were systematically optimized to achieve adequate separation of the 11 selected marker compounds. The final conditions provided good peak resolution, symmetrical peak shapes, and stable retention times for all analytes.

First, four different reversed-phase C_18_ columns—Capcell pak UG80 (Shiseido, Tokyo, Japan), SunFire (Waters, Milford, MA, USA), Gemini (Phenomenex, Torrance, CA, USA), and Hypersil GOLD (Thermo Fisher Scientific, San Jose, CA, USA)—were compared (Figure S3). Among them, the Capcell pak UG80 column provided the best overall separation performance. In contrast, the SunFire column showed insufficient resolution between 4-hydroxycinnamic acid and liquiritin apioside, while the Gemini column exhibited peak overlap with adjacent unknown peaks and peak broadening. The Hypersil GOLD column also resulted in co-elution of liquiritin apioside and liquiritin. Therefore, the Capcell pak UG80 column was selected for further analysis.

The column temperature was then evaluated at 30, 35, 40, and 45 °C (Figure S4). At 30 °C, incomplete separation of 4-hydroxycinnamic acid and liquiritin apioside was observed, whereas at 35 °C, peak overlap between glycyrrhizin and arctigenin occurred. At 45 °C, peak broadening and reduced resolution were observed. In contrast, a temperature of 40 °C provided relatively improved separation and peak shape, and was therefore selected as the optimal column temperature.

The mobile phase composition was further optimized. Initially, water–methanol and water–acetonitrile systems without acidic modifiers were evaluated (Figure S5), but poor resolution and peak overlap were observed. To improve chromatographic performance, several acidic modifiers, including 0.1% formic acid, 0.1% trifluoroacetic acid, 0.1% phosphoric acid, and 1.0% acetic acid, were compared (Figure S6). Among these, the mobile phase containing 0.1% formic acid provided the best peak shape and resolution for all marker compounds.

Based on these results, the optimized HPLC–PDA conditions were established as summarized in Table 1.

### 2.3. Method Validation

The developed HPLC–PDA method was validated in terms of linearity, sensitivity, precision, accuracy, and stability for the simultaneous determination of 11 marker compounds.

Linearity was evaluated using calibration curves constructed from at least six concentration levels for each compound. All calibration curves exhibited excellent linearity with a coefficient of determination (*r*^2^) greater than 0.9999 over the tested concentration ranges (Table 2). The limits of detection (LOD) and limits of quantification (LOQ) ranged from 0.001 to 0.119 μg/mL and 0.003 to 0.360 μg/mL, respectively, indicating the high sensitivity of the method.

The accuracy of the method was evaluated through recovery tests at three concentration levels. The recovery values ranged from 95.64% to 105.69%, with relative standard deviations (RSD) values not exceeding 2.17%, confirming the reliability and accuracy of the method (Table 3).

The precision of the method was assessed by intra- and inter-day analyses. The RSD values for both intra- and inter-day precision were below 2.0% for all analytes, demonstrating good reproducibility (Table 4).

Repeatability and system suitability were also examined. The retention times and peak areas showed low RSD values (less than 0.3%), indicating excellent repeatability based on six replicates (Tables S2 and S3). In addition, most of the system suitability parameters were found to be acceptable; however, the resolution between liquiritin apioside and liquiritin was marginal and was therefore further evaluated by UV spectral matching and peak purity analysis (Table S4).

The stability of the sample solutions was evaluated over 7 days at room temperature. All analytes remained stable, with RSD values below 1.11%, confirming that the sample solutions were sufficiently stable during the analytical period (Table S5).

These results indicate that the developed HPLC–PDA method is reliable, accurate, and suitable for the simultaneous quantification of multiple marker compounds in EGS.

### 2.4. Simultaneous Determination of Marker Compounds

**Figure 1:**
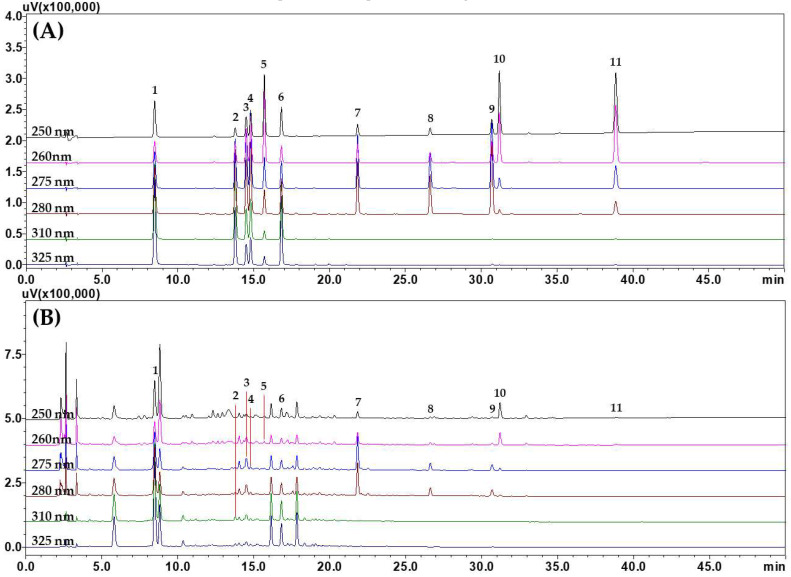
Representative HPLC chromatograms of standard solution (**A**) and EGS–1 sample (**B**). Chlorogenic acid (1), 4-hydroxycinnamic acid (2), liquiritin apioside (3), liquiritin (4), genistin (5), isochlorogenic acid A (6), arctiin (7), matairesinol (8), arctigenin (9), glycyrrhizin (10), and pulegone (11).

The validated HPLC–PDA method was applied to the simultaneous determination of 11 marker compounds in a laboratory-prepared extract (EGS–1) and three commercially available EGS granule products (EGS–2 to EGS–4). Representative chromatograms of the standard mixture and EGS–1 sample are shown in Figure 1, while chromatograms of the commercial products are provided in Figure S7.

As summarized in Table 5, notable differences in the contents of the marker compounds were observed among the samples. The laboratory-prepared extract (EGS–1) exhibited generally higher concentrations of most marker compounds compared to the commercial products (EGS–2 to EGS–4). For instance, chlorogenic acid (6.65 mg/g), arctiin (15.60 mg/g), glycyrrhizin (6.07 mg/g), and matairesinol (5.00 mg/g) were present at relatively high levels in EGS–1.

These differences are likely attributable to variations in formulation. The EGS–1 sample represents a crude extract prepared without excipients, whereas the commercial products (EGS–2 to EGS–4) are granule formulations containing excipients. Such formulation differences can dilute the relative content of marker compounds, resulting in lower apparent concentrations in commercial samples.

In addition to overall differences in concentration, considerable variability among the commercial products (EGS–2 to EGS–4) was observed. For example, chlorogenic acid ranged from 0.24 to 5.35 mg/g, while arctiin varied from 2.15 to 10.79 mg/g. Glycyrrhizin also showed noticeable variation among products.

Furthermore, liquiritin in EGS–2 and 4-hydroxycinnamic acid and liquiritin apioside in EGS–2 to EGS–4 showed peaks at the expected retention times in chromatograms (Figure S7); however, their UV spectral profiles did not match those of the corresponding reference standards (Figure S8). This discrepancy suggests possible co-elution with other components, and therefore these compounds were not quantified because of spectral mismatch (NQ-SM).

These results indicate that the chemical profiles of EGS products vary depending on formulation and manufacturing conditions, highlighting the importance of reliable analytical methods for comprehensive quality evaluation and quality control of multi-herbal formulations.

## 3. Discussion

The development of analytical strategies for multi-herbal formulations requires careful consideration of both chemical representativeness and analytical reliability. In the present study, marker compounds were selected based on their relevance to the constituent herbs and their detectability under chromatographic conditions. This approach reflects a practical compromise between comprehensive phytochemical coverage and methodological robustness, which is widely regarded as essential for quality control of complex herbal medicines [13,29,30]. In particular, the selected compounds encompass diverse chemical classes, including phenolic acids, flavonoids, lignans, and triterpenoid saponins, thereby enabling a multi-dimensional evaluation of the formulation.

The optimization of chromatographic conditions played a critical role in achieving adequate separation of structurally diverse analytes. Differences observed among the tested columns highlight the importance of stationary phase characteristics in herbal analysis. The improved performance of the selected column can be attributed to enhanced selectivity toward polar and structurally similar compounds, such as glycosides and phenolic derivatives, which are abundant in herbal matrices. Similar observations have been reported in previous studies, where column chemistry influenced resolution and peak behavior in multi-component herbal systems [31]. In addition, the incorporation of an acidic modifier in the mobile phase contributed to improved peak symmetry and reproducibility, likely through suppression of secondary interactions and stabilization of analyte ionization, which is consistent with general chromatographic principles for phenolic compounds [32].

The validated analytical method demonstrated adequate performance for simultaneous multi-component quantification, supporting its applicability for routine quality evaluation. In the context of herbal medicine analysis, analytical reliability is particularly important because variability in sample composition can obscure meaningful comparisons if methodological uncertainty is not minimized. The robustness of the method suggests that it can serve as a practical tool for comparative studies of complex formulations, complementing existing chromatographic fingerprinting approaches [29].

Application of the method to different EGS formulations revealed notable variability in chemical composition. Such variability is commonly observed in multi-herbal products and can arise from multiple factors, including differences in raw material origin, harvesting conditions, extraction procedures, and manufacturing processes [10,11]. In addition, formulation-specific factors, such as the inclusion of excipients in granule products, may influence the relative abundance of active constituents. These factors collectively contribute to heterogeneity among products, which has been recognized as a major challenge in the quality control of traditional herbal medicines [10,11].

An important observation in this study was the discrepancy between retention time matching and ultraviolet (UV) spectral consistency for certain peaks. Although chromatographic peaks were observed at expected retention times, their spectral characteristics did not fully correspond to those of reference standards, suggesting the presence of co-eluting components. This limitation is frequently encountered in the analysis of complex herbal matrices using UV-based detection alone, where structurally similar compounds may overlap under chromatographic conditions [15]. Therefore, the integration of complementary analytical techniques, such as liquid chromatography–mass spectrometry, is recommended to enhance selectivity and enable more reliable compound identification [33,34].

The observed variability in marker compound profiles may have important implications for the quality and consistency of EGS products. Since multiple constituents are believed to contribute synergistically to the pharmacological effects of herbal formulations, variations in chemical composition could potentially affect their biological activity and therapeutic efficacy. This underscores the importance of adopting multi-component analytical approaches for quality evaluation, rather than relying on a limited number of marker compounds. The strategy employed in this study provides a more comprehensive framework for assessing the chemical characteristics of multi-herbal formulations and aligns with current trends in chromatographic fingerprinting and quality evaluation of herbal medicines [30].

Despite the strengths of the developed method, certain limitations should be acknowledged. The reliance on UV detection may limit the ability to distinguish structurally similar compounds in highly complex matrices, and the study focused on a limited number of commercial products, which may not fully represent the variability across the broader market. In addition, method validation was performed using the laboratory-prepared EGS extract (EGS–1), and therefore the analytical performance of the method in commercial granule formulations containing different excipients and matrix compositions was not independently validated. Future studies incorporating advanced detection techniques and a larger sample set would further enhance the understanding of chemical variability in EGS formulations.

Overall, the HPLC–PDA method developed in this study provides a reliable and practical analytical platform for the simultaneous determination of multiple marker compounds in EGS. This approach contributes to the advancement of quality control strategies and may facilitate more consistent evaluation of multi-herbal products in both research and industrial settings.

## 4. Materials and Methods

### 4.1. Chemicals and Reagents

Eleven reference standard compounds used as marker analytes for the quality evaluation of EGS were purchased from Merck KGaA (Darmstadt, Germany), Fujifilm Wako Pure Chemical Corporation (Osaka, Japan), Shanghai Sunny Biotech Co., Ltd. (Shanghai, China), and Chengdu Biopurify Phytochemicals Ltd. (Chengdu, China), all with purities ≥ 98.1% (Table S6 and Figure S9). HPLC-grade solvents, including acetonitrile, methanol, and water, were obtained from J.T. Baker (Phillipsburg, NJ, USA). Formic acid, acetic acid (glacial), and phosphoric acid (ACS reagent grade), as well as trifluoroacetic acid (HPLC grade), were purchased from Merck KGaA.

### 4.2. Plant Materials and Samples

The nine herbal materials constituting EGS (Table S7) were purchased in January 2018 from Kwangmyeongdang Pharmaceutical Co. (Ulsan, Republic of Korea). All raw materials were taxonomically authenticated by Dr. Goya Choi at the Korea Institute of Oriental Medicine (KIOM). Voucher specimens (2018–EGS–1 to 2018–EGS–11) were deposited at KIOM.

The laboratory-prepared extract (EGS–1) was prepared by a single extraction of the mixed herbal materials according to the proportions listed in Table S7 (total weight: 5 kg), followed by extraction with ten volumes of distilled water (50 L) at 100 °C for 2 h using a low-temperature vacuum extractor. The extract was filtered and subsequently freeze-dried to yield 998 g of powder (yield: 19.96%). The resulting powder contained no excipients and was stored at −20 °C. Commercial granule formulations (EGS–2 to EGS–4) were purchased from different manufacturers. Detailed product information for the commercial formulations, including country, dosage form, package size, labeled daily dose, batch/lot number, expiry date, and purchase date, is summarized in Table S8.

### 4.3. Preparation of Standard and Sample Solutions

Stock solutions of the 11 reference standards were prepared at 1.0 mg/mL in methanol. Sample solutions were prepared by extracting 100 mg of sample with 10 mL of 70% methanol using ultrasonication for 60 min at room temperature, followed by filtration through a 0.22 μm syringe filter (diameter: 25 mm) (GVS ABLUO, Sandford, ME, USA). The prepared sample solutions were analyzed either directly or after dilution, depending on the concentration levels of the analytes. Specifically, 4-hydroxycinnamic acid, liquiritin, genistin, isochlorogenic acid A, and pulegone were analyzed using the original sample solution, whereas chlorogenic acid, liquiritin apioside, arctiin, matairesinol, arctigenin, and glycyrrhizin were analyzed after 10-fold dilution with 70% methanol. The same dilution procedure was applied to both quantitative analysis and recovery tests.

### 4.4. HPLC–PDA Analysis

HPLC analysis was performed using a Shimadzu Prominence LC-20A system (Tokyo, Japan) equipped with a PDA detector. The system consisted of binary pumps, an online degasser, and an autosampler. Data acquisition and processing were carried out using LCsolution software (version 1.24, Shimadzu). The detailed chromatographic conditions are summarized in Table 1.

### 4.5. Method Validation

The developed HPLC method was validated in accordance with the International Conference on Harmonisation guidelines [35] for linearity, sensitivity (LOD and LOQ), precision (intra- and inter-day), recovery, and stability. All validation procedures were performed using the EGS–1 sample.

Linearity was evaluated using calibration curves constructed for each marker compound, expressed as *y* = a*x* + b, where y represents the peak area and x represents the concentration (μg/mL) of the reference standard. The *r*^2^ value was used to assess linearity.

Sensitivity was determined by calculating the LOD and LOQ using the following equations: LOD = 3.3*σ*/*S* and LOQ = 10*σ*/*S*, where *σ* represents the standard deviation of the y-intercept and *S* is the slope of the calibration curve.

Recovery was evaluated using the standard addition method by spiking the EGS sample with known amounts of reference standards at three levels (80%, 100%, and 120%). Recovery (%) was calculated as follows: (detected amount − original amount)/spiked amount × 100.

Precision was assessed based on RSD values obtained from intra- and inter-day analyses.

Stability was evaluated by analyzing the sample solution over a period of 7 days at room temperature. The initial measurement was set as 100%, and variations in each marker compound were monitored over time.

Peak identification was performed based on retention time and comparison of UV spectra with those of the corresponding reference standards. Peaks showing spectral mismatch were not quantified.

## 5. Conclusions

In this study, a reliable and validated HPLC–PDA method was developed for the simultaneous determination of 11 marker compounds in EGS. The method demonstrated excellent linearity, sensitivity, accuracy, and precision, confirming its suitability for quantitative analysis. The validated method was successfully applied to the comparative analysis of a laboratory-prepared extract and commercially available formulations, revealing notable differences in the chemical profiles of the samples. These results indicate that the tested EGS products exhibited differences in marker profiles and relative compound contents, potentially reflecting differences in formulation and manufacturing conditions. Overall, the proposed analytical method provides a practical and effective approach for the comparative quality evaluation and quality control of EGS formulations and may serve as a useful analytical tool for the evaluation of complex herbal formulations.

## Figures and Tables

**Table 1:** HPLC operating conditions for the simultaneous determination of 11 marker compounds in Eungyosan.

Parameters for Simultaneous Analysis of 11 Marker Compounds			
Column	Capcell pak UG80 C_18_ (250 mm × 4.6 mm, 5 μm)		
Detector	Photodiode array (monitoring at 250, 260, 275, 280, 310, and 325 nm)		
Flow rate (mL/min)	1		
Injection volume (μL)	10		
Column temperature (°C)	40		
Mobile phase	Solvent A: 0.1% (*v*/*v*) formic acid in distilled water Solvent B: 0.1% (*v*/*v*) formic acid in acetonitrile		
Gradient flow	Time (min)	Solvent A (%)	Solvent B (%)
	0	90	10
	40	40	60
	50	40	60
	60	90	10
	70	90	10

**Table 2:** Regression equation, linearity, and sensitivity data for the marker compounds.

Compound	Detection Wavelength (nm)	Linear Range (μg/mL)	Regression Equation	Coefficient of Determination	LOD (μg/mL) ^1^	LOQ (μg/mL) ^2^
Chlorogenic acid	325	0.31–20.00	*y* = 40,905.34*x* + 527.99	1.0000	0.012	0.037
4-Hydroxycinnamic acid	310	0.31–20.00	*y* = 93,596.18*x* + 2942.11	1.0000	0.007	0.021
Liquiritin apioside	275	0.31–20.00	*y* = 15,476.71*x* + 350.93	1.0000	0.026	0.079
Liquiritin	275	0.31–20.00	*y* = 24,115.92*x* + 154.55	1.0000	0.019	0.058
Genistin	260	0.31–20.00	*y* = 48,176.96*x* + 1339.87	1.0000	0.001	0.003
Isochlorogenic acid A	325	0.78–50.00	*y* = 39,164.40*x* + 1791.93	1.0000	0.055	0.167
Arctiin	275	0.78–50.00	*y* = 6189.33*x* + 362.21	1.0000	0.119	0.360
Matairesinol	280	0.31–20.00	*y* = 5154.69*x* − 660.01	1.0000	0.070	0.213
Arctigenin	280	0.31–20.00	*y* = 9613.22*x* + 93.18	1.0000	0.023	0.069
Glycyrrhizin	250	0.78–50.00	*y* = 8151.16*x* + 543.81	1.0000	0.025	0.076
Pulegone	250	0.78–50.00	*y* = 21,289.89*x* + 1846.39	1.0000	0.039	0.117

^1^ LOD: limit of detection. ^2^ LOQ: limit of quantification.

**Table 3:** Recovery (%) of the selected 11 marker compounds in the established HPLC–PDA method.

Compound	Original Amount (μg/mL) ^1^	Spiked Amount (μg/mL)	Detected Amount (μg/mL)	Recovery (%) ^2^	SD ^3^	RSD (%) ^4^
Chlorogenic acid	6.66	5.00	11.80	102.75	1.21	1.17
		6.00	12.97	105.24	1.01	0.96
		8.00	15.08	105.23	2.07	1.97
4-Hydroxycinnamic acid	1.93	1.50	3.44	101.11	0.92	0.91
		2.00	3.96	101.45	0.98	0.97
		2.40	4.41	103.55	0.88	0.85
Liquiritin apioside	2.49	2.00	4.58	104.51	0.58	0.55
		2.50	5.01	100.60	0.11	0.11
		3.00	5.49	99.76	0.90	0.90
Liquiritin	6.73	4.00	10.88	103.81	2.26	2.17
		6.00	12.88	102.43	0.87	0.85
		8.00	15.02	103.62	1.32	1.27
Genistin	3.44	3.00	6.57	104.39	1.07	1.02
		3.50	6.98	101.01	0.73	0.72
		4.50	8.05	102.35	0.95	0.93
Isochlorogenic acid A	18.92	15.00	34.02	100.67	0.88	0.88
		20.00	39.05	100.64	0.62	0.61
		24.00	43.07	100.60	0.39	0.38
Arctiin	15.75	12.00	28.01	102.11	0.81	0.79
		15.00	31.61	105.69	1.33	1.26
		18.00	34.63	104.87	1.88	1.79
Matairesinol	5.35	4.00	9.43	102.07	1.81	1.77
		5.00	10.46	102.34	1.91	1.87
		6.00	11.53	102.98	1.56	1.51
Arctigenin	2.62	2.00	4.66	102.04	0.47	0.46
		2.50	5.05	97.49	1.44	1.48
		3.00	5.61	99.81	0.87	0.87
Glycyrrhizin	6.21	5.00	11.33	102.43	0.67	0.65
		6.00	12.47	104.30	0.92	0.89
		7.00	13.52	104.46	0.93	0.89
Pulegone	1.76	1.50	3.20	95.64	0.76	0.80
		1.80	3.51	97.12	0.98	1.01
		2.00	3.70	96.78	0.93	0.97

^1^ “Original amount” values represent the measured concentrations in the injected solutions after dilution, when applicable. ^2^ Recovery (%) = [(detected amount − original amount)/spiked amount] × 100. ^3^ SD: standard deviation. ^4^ RSD: relative standard deviation.

**Table 4:** Precision test of 11 marker compounds in the established HPLC–PDA method.

Compound	Concentration (μg/mL)	Intra-Day (n = 5)				Inter-Day (n = 15)	
		Found Concentration. (μg/mL)	Precision (RSD, %)	Accuracy (%)	Found Concentration (μg/mL)	Precision (RSD, %)	Accuracy (%)
Chlorogenic acid	0.31	0.30	0.92	97.07	0.30	1.77	97.70
	2.50	2.54	0.35	101.64	2.57	1.04	102.79
	20.00	19.77	0.50	98.85	20.03	1.45	100.14
4-Hydroxycinnamic acid	0.31	0.29	1.17	94.58	0.29	0.79	94.54
	2.50	2.55	0.52	102.12	2.58	0.95	103.23
	20.00	19.81	0.44	99.03	20.04	1.18	100.20
Liquiritin apioside	0.31	0.31	1.10	99.68	0.31	1.90	98.54
	2.50	2.54	0.33	101.78	2.57	1.31	102.90
	20.00	19.80	0.51	99.02	20.10	1.59	100.50
Liquiritin	0.31	0.31	0.63	101.21	0.31	1.38	100.52
	2.50	2.53	0.31	101.36	2.56	1.29	102.53
	20.00	19.82	0.66	99.12	20.10	1.57	100.52
Genistin	0.31	0.30	1.17	96.45	0.30	1.16	95.76
	2.50	2.54	0.50	101.71	2.57	1.09	102.85
	20.00	19.80	0.46	99.01	20.10	1.63	100.52
Isochlorogenic acid A	0.78	0.76	0.97	97.52	0.76	0.81	97.31
	6.25	6.38	0.36	102.06	6.45	1.04	103.12
	50.00	49.45	0.39	98.90	49.99	1.07	99.98
Arctiin	0.78	0.76	1.12	97.80	0.77	1.84	98.14
	6.25	6.35	0.70	101.65	6.44	1.30	103.01
	50.00	49.41	0.47	98.82	50.15	1.65	100.30
Matairesinol	0.31	0.33	1.61	107.49	0.33	1.54	106.96
	2.50	2.53	0.52	101.14	2.55	1.14	102.12
	20.00	19.91	0.67	99.54	20.17	1.23	100.83
Arctigenin	0.31	0.32	1.01	103.88	0.32	1.79	103.01
	2.50	2.55	0.75	101.99	2.56	1.27	102.33
	20.00	19.84	0.53	99.20	20.09	1.27	100.43
Glycyrrhizin	0.78	0.77	1.23	98.24	0.77	0.94	98.39
	6.25	6.37	0.45	101.85	6.42	0.90	102.70
	50.00	49.53	0.49	99.05	50.19	1.53	100.38
Pulegone	0.78	0.74	0.48	95.06	0.74	0.72	94.86
	6.25	6.37	0.59	101.94	6.44	1.07	103.12
	50.00	49.81	0.46	99.63	50.12	0.55	100.25

**Table 5:** Quantitative analysis of 11 marker compounds in EGS samples by the validated HPLC–PDA method.

Compound	Sample											
	EGS–1			EGS–2			EGS–3			EGS–4		
	Mean (mg/g)	SD	RSD (%)	Mean (mg/g)	SD	RSD (%)	Mean (mg/g)	SD	RSD (%)	Mean (mg/g)	SD	RSD (%)
Chlorogenic acid	6.65	0.02	0.33	0.24	0.00	0.18	0.88	0.01	0.94	5.35	0.01	0.10
4-Hydroxycinnamic acid	0.20	0.00	0.45	NQ-SM ^1^	–	–	NQ-SM	–	–	NQ-SM	–	–
Liquiritin apioside	2.47	0.02	0.87	NQ-SM	–	–	NQ-SM	–	–	NQ-SM	–	–
Liquiritin	0.68	0.01	1.36	NQ-SM	–	–	0.49	0.00	0.56	2.75	0.02	0.75
Genistin	0.41	0.00	1.04	0.04	0.00	2.00	0.19	0.00	0.22	ND	–	–
Isochlorogenic acid A	1.96	0.02	0.85	0.42	0.01	1.23	0.34	0.00	0.11	1.76	0.00	0.26
Arctiin	15.60	0.05	0.33	2.15	0.01	0.45	5.04	0.04	0.71	10.79	0.10	0.89
Matairesinol	5.00	0.05	1.06	0.06	0.00	2.15	0.14	0.00	0.98	0.60	0.01	1.55
Arctigenin	2.55	0.02	0.97	0.15	0.00	1.47	0.42	0.01	1.37	1.00	0.01	0.52
Glycyrrhizin	6.07	0.09	1.45	2.23	0.01	0.39	3.42	0.02	0.46	6.61	0.03	0.41
Pulegone	0.18	0.00	0.47	ND ^2^	–	–	ND	–	–	0.24	0.00	0.49

Reported contents were calculated as C × V × D/m, where C is the measured concentration in the injected solution, V is extraction volume, D is dilution factor, and m is sample mass. ^1^ NQ-SM: not quantified due to spectral mismatch. ^2^ ND: not detected (below the limit of detection).

## Data Availability

All data in this study can be found in this paper.

## Supplementary Materials

The following supporting information can be downloaded at https://www.mdpi.com/article/10.3390/molecules31121991/s1, Figure S1: HPLC profiles of the nine raw herbal medicines and their major components. (A): *F. viridissima*; (B): *L. japonica*; (C): *P. grandiflorum*; (D): *M. arvensis*; (E): *L. gracile*; (F): *G. uralensis*; (G): *S. tenuifolia*; (H): *G. max*; and (I): *A. lappa*; Figure S2: HPLC chromatograms of a standard solution containing 24 reference standards (A) and 70% methanol solution of Eungyosan water extract (B) monitored at various wavelengths. Chlorogenic acid (1), homoorientin (2), daidzin (3), glycitin (4), orientin (5), 4-hydroxycinnamic acid (6), vitexin (7), liquiritin apioside (8), liquiritin (9), genistin (10), malonyldaidzin (11), isochlorogenic acid A (12), malonylgenistin (13), arctiin (14), daidzein (15), platycodin D2 (16), platycodin D (17), liquiritigenin (18), matairesinol (19), menthone (20), arctigenin (21), glycyrrhizin (22), menthol (23), and pulegone (24); Figure S3: Comparison of HPLC chromatograms of marker compounds using different C_18_ columns. (A): Capcell pak UG80, (B): SunFire™, (C): Gemini, and (D): Hypersil GOLD. Chlorogenic acid (1), 4-hydroxycinnamic acid (2), liquiritin apioside (3), liquiritin (4), genistin (5), isochlorogenic acid A (6), arctiin (7), matairesinol (8), arctigenin (9), glycyrrhizin (10), and pulegone (11); Figure S4: Comparison of HPLC chromatograms of marker compounds at different column oven temperatures. (A): 30 °C, (B): 35 °C, (C): 40 °C and (D): 45 °C. Chlorogenic acid (1), 4-hydroxycinnamic acid (2), liquiritin apioside (3), liquiritin (4), genistin (5), isochlorogenic acid A (6), arctiin (7), matairesinol (8), arctigenin (9), glycyrrhizin (10), and pulegone (11); Figure S5: Comparison of HPLC chromatograms of marker compounds using different solvent systems. (A): water–methanol system and (B): water–acetonitrile system. Chlorogenic acid (1), 4-hydroxycinnamic acid (2), liquiritin apioside (3), liquiritin (4), genistin (5), isochlorogenic acid A (6), arctiin (7), matairesinol (8), arctigenin (9), glycyrrhizin (10), and pulegone (11); Figure S6: Comparison of HPLC chromatograms of marker compounds using different acidic modifiers in the mobile phase. (A): 0.1% (*v*/*v*) formic acid, (B): 0.1% (*v*/*v*) trifluoroacetic acid, (C): 0.1% (*v*/*v*) phosphoric acid, and (D): 1.0% (*v*/*v*) acetic acid. Chlorogenic acid (1), 4-hydroxycinnamic acid (2), liquiritin apioside (3), liquiritin (4), genistin (5), isochlorogenic acid A (6), arctiin (7), matairesinol (8), arctigenin (9), glycyrrhizin (10), and pulegone (11); Figure S7: HPLC chromatograms of the commercial EGS formulations. (A): EGS–2, (B): EGS–3, and (C): EGS–4. Chlorogenic acid (1), 4-hydroxycinnamic acid (2), liquiritin apioside (3), liquiritin (4), genistin (5), isochlorogenic acid A (6), arctiin (7), matairesinol (8), arctigenin (9), glycyrrhizin (10), and pulegone (11); Figure S8: Comparison of UV spectral profiles between selected peaks in commercial EGS formulations and corresponding reference standards. (A): EGS–2, (B): EGS–3, and (C): EGS–4; Figure S9: Chemical structures of 11 analytes selected as marker compounds for quality control of Eungyosan; Table S1: Candidate and selected marker compounds investigated for EGS analysis and reasons for selection or exclusion; Table S2: Retention time reproducibility of 11 marker analytes; Table S3: Peak area reproducibility of 11 marker analytes; Table S4: System suitability parameters for 11 marker analytes; Table S5: Stability evaluation of 11 marker analytes; Table S6: Reference standard compounds selected as marker analytes for quality evaluation of Eungyosan; Table S7: Composition of Eungyosan; Table S8: Product information for commercially available EGS formulations analyzed in this study.

## Abbreviations

The following abbreviations are used in this manuscript:

EGS	Eungyosan
HPLC	High-performance liquid chromatography
KIOM	Korea Institute of Oriental Medicine
LOD	Limits of detection
LOQ	Limits of quantification
*N*	Theoretical plate number
ND	Not detected
NQ-SM	Not quantified due to spectral mismatch
PDA	Photodiode array detector
*r*^2^	Coefficient of determination
*Rs*	Resolution
RSD	Relative standard deviation
*S*	Symmetry factor
UV	Ultraviolet
