# Supplementary material for: Quality Comparative Evaluation of Eungyosan Formulations by a Validated HPLC–PDA Method for 11 Marker Components
Source: Molecules. 2026 Jun 7;31(12):1991. doi: 10.3390/molecules31121991 (PMC13304645; doi:10.3390/molecules31121991)
Supplement: Supplementary file 1 [file molecules-31-01991-s001.zip › molecules-4349043-SI.pdf]

Supplementary Materials

Quality Comparative Evaluation of Eungyosan Formulations by a Validated HPLC–PDA Method for 11 Marker Components

Table S1

Candidate and selected marker compounds investigated for EGS analysis and reasons for selection or exclusion.

Compound	Representative source herb	Detection wavelength (nm)	Final selection	Reason for selection/exclusion
Chlorogenic acid	*Lonicera japonica*<br>*Arctium lappa*<br>*Lophatherum gracile*	325	Yes	Consistently detected with stable and reproducible chromatographic peaks in EGS
Arctiin	*Forsythia viridissima*<br>*Arctium lappa*	280	Yes	
Arctigenin	*Forsythia viridissima*<br>*Arctium lappa*	280	Yes	
Matairesinol	*Forsythia viridissima*<br>*Arctium lappa*	280	Yes	
4-Hydroxycinnamic acid	*Lophatherum gracile*	310	Yes	
Liquiritin apioside	*Glycyrrhiza uralensis*	275	Yes	
Liquiritin	*Glycyrrhiza uralensis*	275	Yes	
Glycyrrhizin	*Glycyrrhiza uralensis*	250	Yes	
Genistin	*Glycine max*	260	Yes	
Isochlorogenic acid A	*Lonicera japonica*	325	Yes	
Pulegone	*Schizonepeta tenuifolia*	250	Yes	
Homoorientin	*Lophatherum gracile*	350	No	Not detected or not consistently detected in EGS under the established HPLC–PDA conditions
Orientin	*Lophatherum gracile*	350	No	
Vitexin	*Lophatherum gracile*	335	No	
Daidzin	*Glycine max*	250	No	
Glycitin	*Glycine max*	255	No	
Malonyldaidzin	*Glycine max*	250	No	
Malonylgenistin	*Glycine max*	260	No	
Daidzein	*Glycine max*	250	No	

Platycodin D2	*Platycodon grandiflorum*	203	No
Platycodin D	*Platycodon grandiflorum*	203	No
Liquiritigenin	*Glycyrrhiza uralensis*	275	No
Menthone	*Mentha arvensis*	203	No
Menthol	*Mentha arvensis*	203	No

Table S2

Retention time reproducibility of 11 marker analytes.

Analyte[1]	No. (#), retention time (min)						Mean	SD[2]	RSD (%)[3]
	1	2	3	4	5	6			
1	8.461	8.465	8.458	8.463	8.463	8.457	8.461	0.003	0.037
2	13.775	13.781	13.772	13.777	13.777	13.770	13.775	0.004	0.029
3	14.498	14.501	14.493	14.502	14.498	14.491	14.497	0.004	0.030
4	14.792	14.795	14.789	14.796	14.792	14.786	14.792	0.004	0.025
5	15.695	15.699	15.695	15.699	15.694	15.692	15.696	0.003	0.018
6	16.819	16.825	16.821	16.822	16.819	16.819	16.821	0.002	0.014
7	21.840	21.841	21.840	21.842	21.838	21.835	21.839	0.003	0.011
8	26.630	26.633	26.629	26.631	26.631	26.624	26.630	0.003	0.012
9	30.696	30.701	30.698	30.699	30.698	30.692	30.697	0.003	0.010
10	31.194	31.200	31.197	31.199	31.199	31.191	31.197	0.004	0.011
11	38.872	38.876	38.872	38.874	38.876	38.866	38.873	0.004	0.010

[1] Analytes: chlorogenic acid (1), 4-hydroxycinnamic acid (2), liquiritin apioside (3), liquiritin (4), genistin (5), isochlorogenic acid A (6), arctiin (7), matairesinol (8), arctigenin (9), glycyrrhizin (10), and pulegone (11).

[2] SD: standard deviation.

[3] RSD: relative standard deviation.

Table S3

Peak area reproducibility of 11 marker analytes.

Analyte[1]	No. (#), peak area						Mean	SD	RSD (%)
	1	2	3	4	5	6			
1	1,228,634	1,228,267	1,230,078	1,232,494	1,234,030	1,233,684	1,231,197.83	2,541.63	0.21
2	1,115,045	1,116,054	1,115,899	1,117,430	1,118,305	1,119,479	1,117,035.33	1,670.63	0.15
3	772,670	772,129	771,005	774,363	776,012	774,551	773,455.00	1,840.70	0.24
4	968,364	969,614	969,531	971,870	971,491	973,200	970,678.33	1,802.48	0.19
5	845,869	850,831	846,647	847,708	851,440	850,622	848,852.83	2,400.67	0.28
6	802,628	805,548	804,240	805,444	805,652	807,548	805,176.67	1,639.00	0.20
7	637,542	639,186	639,900	637,477	639,544	640,246	638,982.50	1,194.70	0.19
8	532,455	533,170	533,572	534,402	533,822	534,829	533,708.33	851.96	0.16
9	1,019,524	1,021,127	1,021,780	1,022,615	1,023,561	1,024,351	1,022,159.67	1,739.56	0.17
10	824,921	827,034	827,901	828,308	829,619	829,306	827,848.17	1,715.02	0.21
11	1,057,606	1,057,626	1,058,458	1,059,390	1,059,763	1,059,938	1,058,796.83	1,047.98	0.10

[1] Analytes: chlorogenic acid (1), 4-hydroxycinnamic acid (2), liquiritin apioside (3), liquiritin (4), genistin (5), isochlorogenic acid A (6), arctiin (7), matairesinol (8), arctigenin (9), glycyrrhizin (10), and pulegone (11).

Table S4
System suitability parameters for 11 marker analytes.

Analyte[1]	Parameter[2]		
	N	*Rs*	*S*
1	61027.03	24.88	1.15
2	229565.97	3.55	1.14
3	215236.61	1.45	1.25
4	264714.72	1.45	1.15
5	344250.89	4.80	1.18
6	380949.43	6.14	1.16
7	619411.54	26.93	1.16
8	703485.05	18.38	1.11
9	839602.95	2.30	1.10
10	1046025.95	31.14	1.19
11	846530.66	31.14	1.06

[1] Analytes: chlorogenic acid (1), 4-hydroxycinnamic acid (2), liquiritin apioside (3), liquiritin (4), genistin (5), isochlorogenic acid A (6), arctiin (7), matairesinol (8), arctigenin (9), glycyrrhizin (10), and pulegone (11).
[2] Parameters: *N*; theoretical plate number, *Rs*; resolution, and *S*; symmetry factor.
Rs values were calculated based on the resolution between adjacent peaks.

Table S5

Stability evaluation of 11 marker analytes.

Analyte[1]	Day							Mean (%)	SD	RSD (%)
	0	0.5	1	2	3	4	7			
1	100.00	100.66	100.22	101.48	98.51	99.46	99.16	99.93	0.99	0.99
2	100.00	99.77	101.49	101.54	100.93	101.60	101.26	100.94	0.76	0.75
3	100.00	100.42	99.61	99.32	98.84	99.13	99.00	99.48	0.57	0.57
4	100.00	101.04	99.48	100.78	100.58	99.10	97.99	99.85	1.08	1.08
5	100.00	100.26	100.41	100.17	99.07	99.55	99.80	99.89	0.47	0.47
6	100.00	100.37	99.49	99.45	99.55	100.33	100.14	99.90	0.40	0.40
7	100.00	100.76	100.12	99.38	99.55	100.11	99.42	99.90	0.50	0.50
8	100.00	98.58	98.72	98.40	99.00	99.18	99.20	99.01	0.53	0.53
9	100.00	99.09	97.62	97.72	98.54	98.26	98.60	98.55	0.82	0.83
10	100.00	100.10	99.93	98.28	97.24	98.83	98.29	98.96	1.09	1.11
11	100.00	99.75	99.70	99.45	99.53	97.09	98.46	99.14	1.03	1.04

[1] Analytes: chlorogenic acid (1), 4-hydroxycinnamic acid (2), liquiritin apioside (3), liquiritin (4), genistin (5), isochlorogenic acid A (6), arctiin (7), matairesinol (8), arctigenin (9), glycyrrhizin (10), and pulegone (11).

Table S6

Reference standard compounds selected as marker analytes for quality evaluation of Eungyosan.

Analyte[1]	Purity (%)	Molecular formula	Molecular weight (g/mol)	CAS No.	PubChem CID	Catalog No.	Maker
1	99.7	$C_{16}H_{18}O_9$	354.31	327-97-9	1794427	PHL89175	Merck KGaA
2	99.2	$C_9H_8O_3$	164.16	501-98-4	637542	082-06521	Fujifilm Wako Pure Chemical Corporation
3	99.6	$C_{26}H_{30}O_{13}$	550.51	74639-14-8	10076238	DR10690	Shanghai Sunny Biotech Co., Ltd.
4	99.6	$C_{21}H_{22}O_9$	418.40	551-15-5	503737	BP0874	Chengdu Biopurify Phytochemicals Ltd.
5	98.3	$C_{21}H_{20}O_{10}$	432.38	52-59-9	5281377	BP0635	Chengdu Biopurify Phytochemicals Ltd.
6	98.2	$C_{25}H_{24}O_{12}$	516.45	2450-53-5	6474310	DR11570	Shanghai Sunny Biotech Co., Ltd.
7	98.1	$C_{27}H_{34}O_{11}$	534.56	20362-31-6	100528	DR11011	Shanghai Sunny Biotech Co., Ltd.
8	98.3	$C_{20}H_{22}O_6$	358.39	580-72-3	119205	DR11516	Shanghai Sunny Biotech Co., Ltd.
9	99.4	$C_{21}H_{24}O_6$	372.42	7770-78-7	64981	DR11012	Shanghai Sunny Biotech Co., Ltd.
10	99.1	$C_{42}H_{62}O_{16}$	822.94	1405-86-3	14982	BP0682	Chengdu Biopurify Phytochemicals Ltd.
11	99.3	$C_{10}H_{16}O$	152.24	89-82-7	442495	BPF3286	Chengdu Biopurify Phytochemicals Ltd.

[1] Analytes: chlorogenic acid (1), 4-hydroxycinnamic acid (2), liquiritin apioside (3), liquiritin (4), genistin (5), isochlorogenic acid A (6), arctiin (7), matairesinol (8), arctigenin (9), glycyrrhizin (10), and pulegone (11).

Table S7

Composition of Eungyosan.

Herbal name	Scientific name	Family	Plant part used	Origin	Amount (g)	Ratio (%)
Forsythiae Fructus	*Forsythia viridissima* Lindl.	Oleaceae	Fruit	Uiseong, Korea	892.9	17.9
Lonicerae Flos	*Lonicera japonica* Thunb.	Caprifoliaceae	Flower buds	China	892.9	17.9
Platycodonis Radix	*Platycodon grandiflorum* A.DC.	Campanulaceae	Root	Yeongju, Korea	535.7	10.7
Menthae Herba	*Mentha arvensis* L.	Lamiaceae	Aerial parts	Uiseong, Korea	535.7	10.7
Lophatheri Herba	*Lophatherum gracile* Brongn.	Poaceae	Aerial parts	Yeongcheon, Korea	357.1	7.1
Glycyrrhizae Radix et Rhizoma Preparata cum Melle	*Glycyrrhiza uralensis* Fisch.	Fabaceae	Root and rhizome	China	446.4	8.9
Schizonepetae Spica	*Schizonepeta tenuifolia* Briq.	Lamiaceae	Spike inflorescence	Yeongcheon, Korea	357.1	7.1
Glycine Semen Preparata	*Glycine max* (L.) Merr.	Leguminosae	Fermented mature seeds	China	446.4	8.9
Arctii Fructus	*Arctium lappa* L.	Asteraceae	Fruit	China	535.7	10.7
				Total	5,000.0	100.0

Table S8

Product information for commercially available EGS formulations analyzed in this study.

Item	EGS–2	EGS–3	EGS–4
Manufacturer code	Manufacturer A	Manufacturer B	Manufacturer C
Country	Korea	Korea	Japan
Dosage form	Granules	Granules	Stick granules
Package size	2.3 g × 120 packets	2.3 g × 120 packets	2.3 g × 9 sticks
Labeled daily dose	3 packets/day	3 packets/day	3 packets/day
Batch/Lot No.	2007	22001	19P519
Expiry date	2023.12	2025.03	2026.09
Purchase date	2022.03	2022.03	2022.03

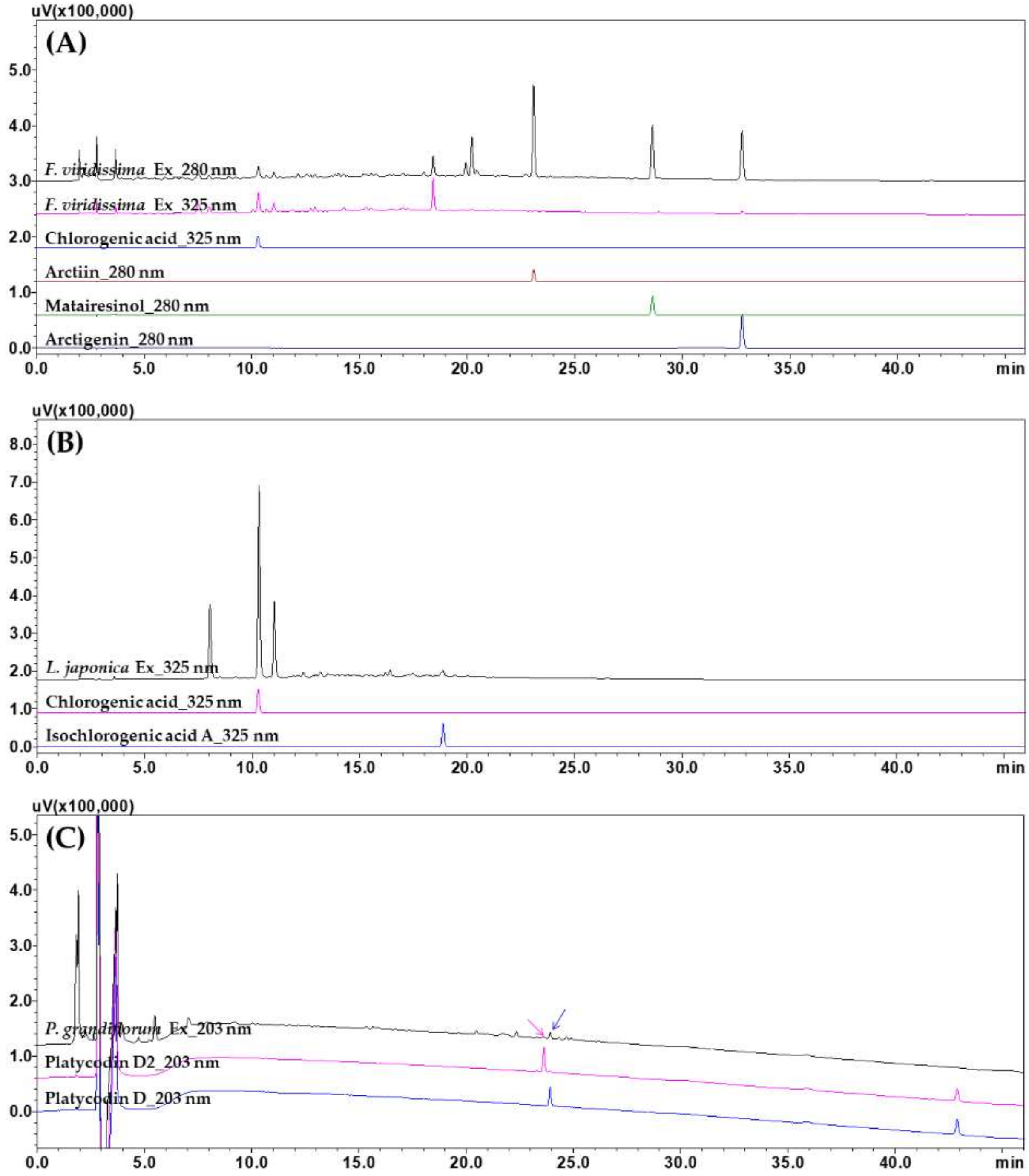
uV(x100,000)
(A)
F. viridissima Ex_280 nm
F. viridissima Ex_325 nm
Chlorogenic acid_325 nm
Arctiin_280 nm
Matairesinol_280 nm
Arctigenin_280 nm
min
(B)
L. japonica Ex_325 nm
Chlorogenic acid_325 nm
Isochlorogenic acid A_325 nm
(C)
P. grandiflorum Ex_203 nm
Platycodin D2_203 nm
Platycodin D_203 nm

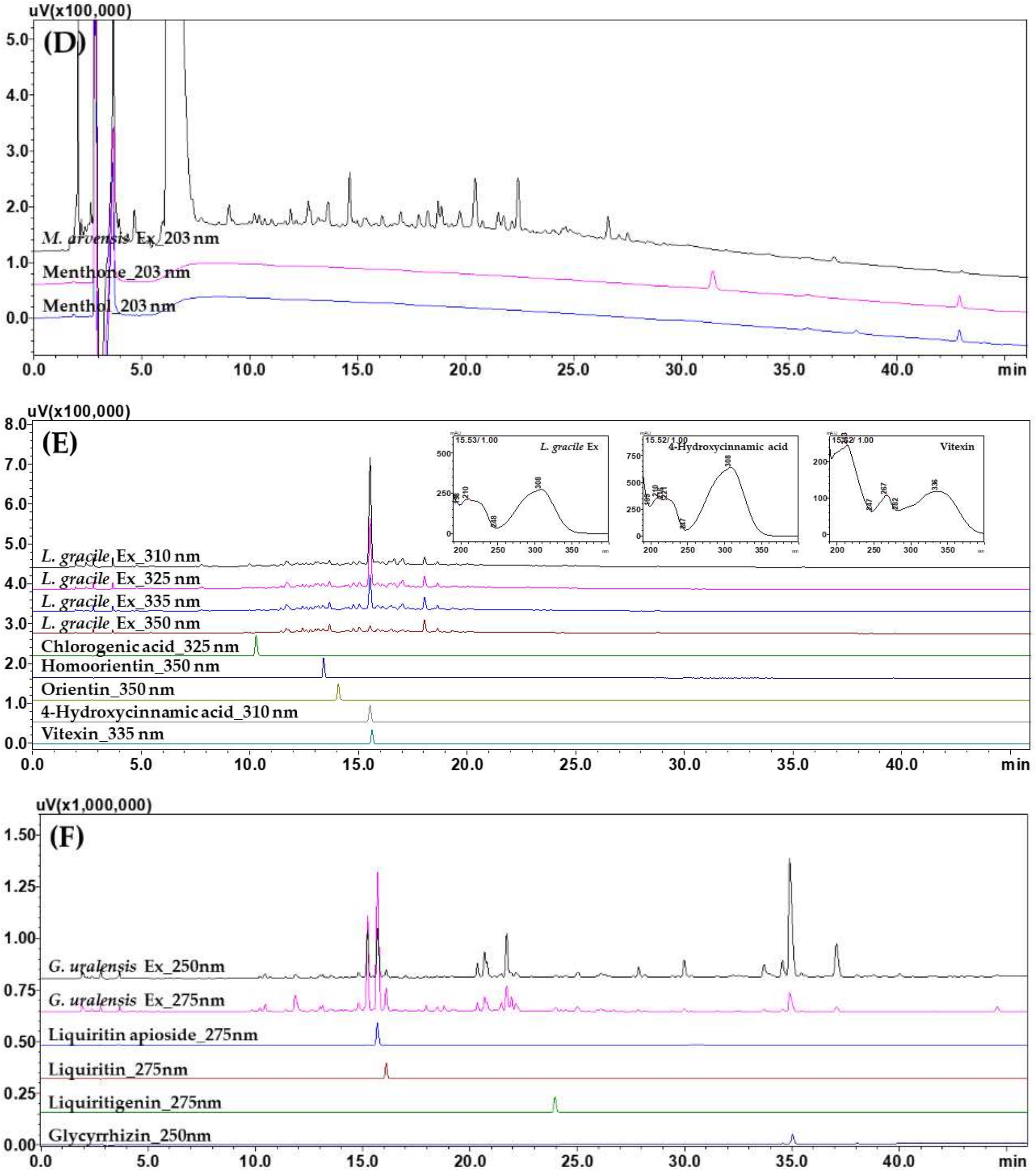
uV(x100,000)
(D)
M. arvensis Ex_203 nm
Menthone_203 nm
Menthol_203 nm
min
uV(x100,000)
(E)
L. gracile Ex
4-Hydroxycinnamic acid
Vitexin
L. gracile Ex_310 nm
L. gracile Ex_325 nm
L. gracile Ex_335 nm
L. gracile Ex_350 nm
Chlorogenic acid_325 nm
Homoorientin_350 nm
Orientin_350 nm
4-Hydroxycinnamic acid_310 nm
Vitexin_335 nm
min
uV(x1,000,000)
(F)
G. uralensis Ex_250nm
G. uralensis Ex_275nm
Liquiritin apioside_275nm
Liquiritin_275nm
Liquiritigenin_275nm
Glycyrrhizin_250nm
min

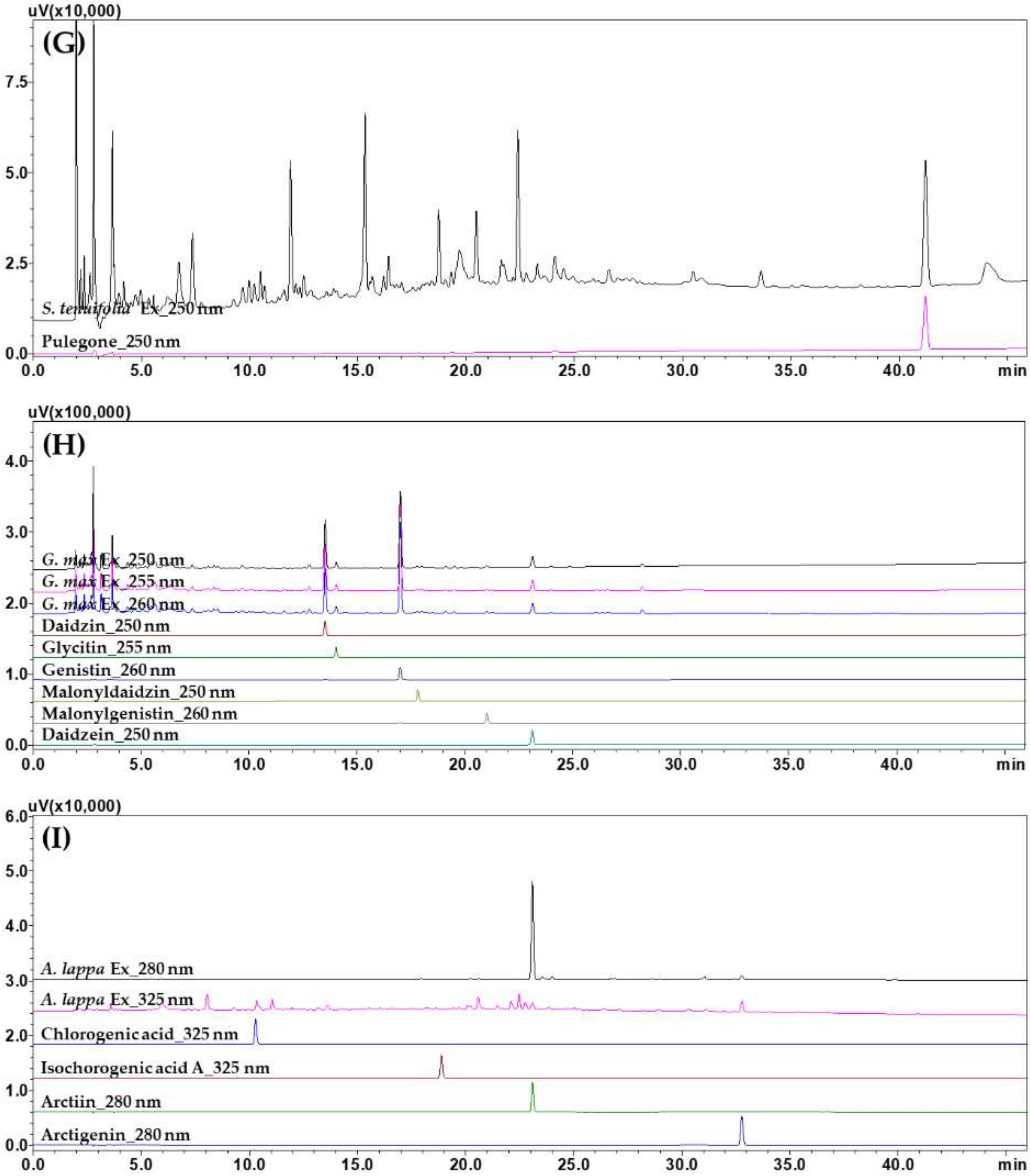

Figure S1. HPLC profiles of the nine raw herbal medicines and their major components. A: *F. viridissima*; B: *L. japonica*; C: *P. grandiflorum*; D: *M. arvensis*; E: *L. gracile*; F: *G. uralensis*; G: *S. tenuifolia*; H: *G. max*; and I: *A. lappa*.

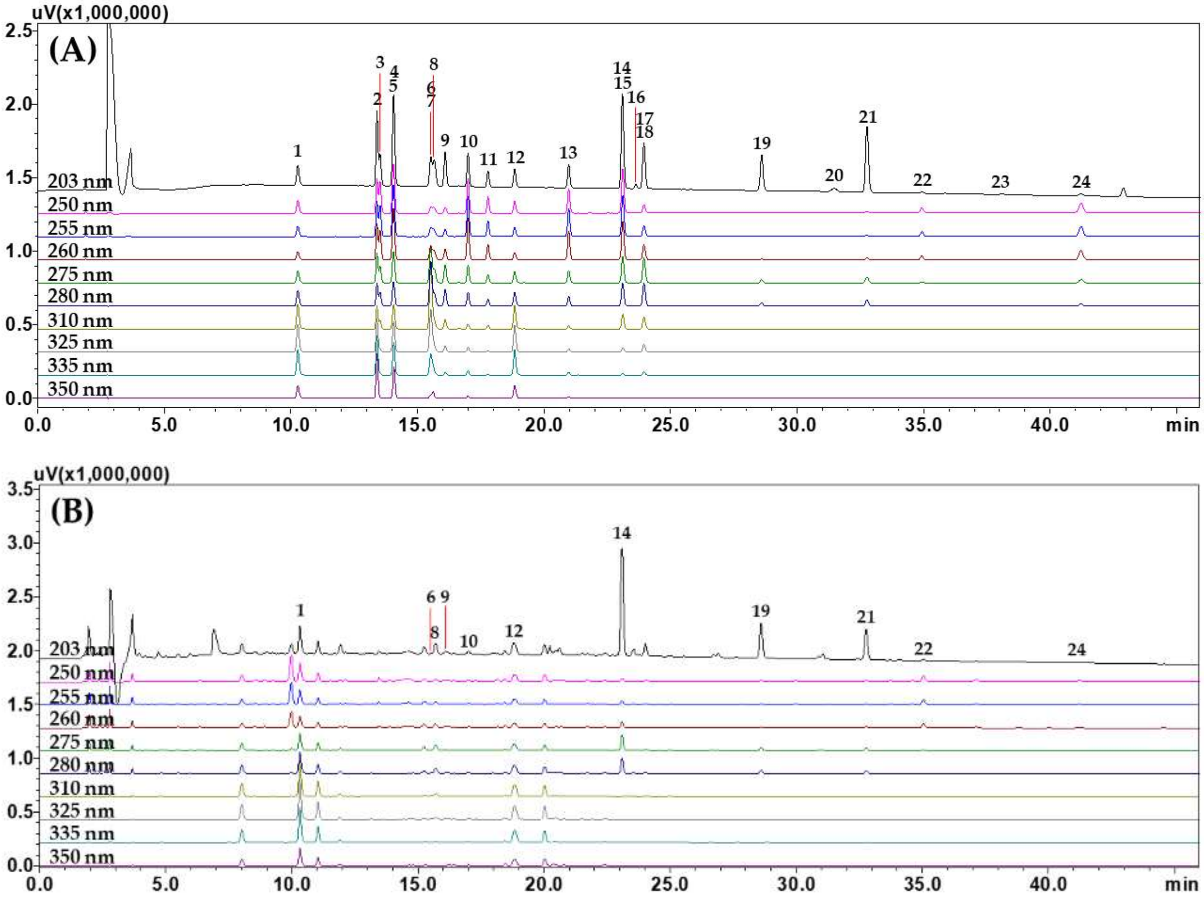

Figure S2. HPLC chromatograms of a standard solution containing 24 reference standards (A) and 70% methanol solution of Eungyosan water extract (B) monitored at various wavelengths. Chlorogenic acid (1), homoorientin (2), daidzin (3), glycitin (4), orientin (5), 4-hydroxycinnamic acid (6), vitexin (7), liquiritin apioside (8), liquiritin (9), genistin (10), malonyldaidzin (11), isochlorogenic acid A (12), malonylgenistin (13), arctiin (14), daidzein (15), platycodin D2 (16), platycodin D (17), liquiritigenin (18), matairesinol (19), menthone (20), arctigenin (21), glycyrrhizin (22), menthol (23), and pulegone (24).

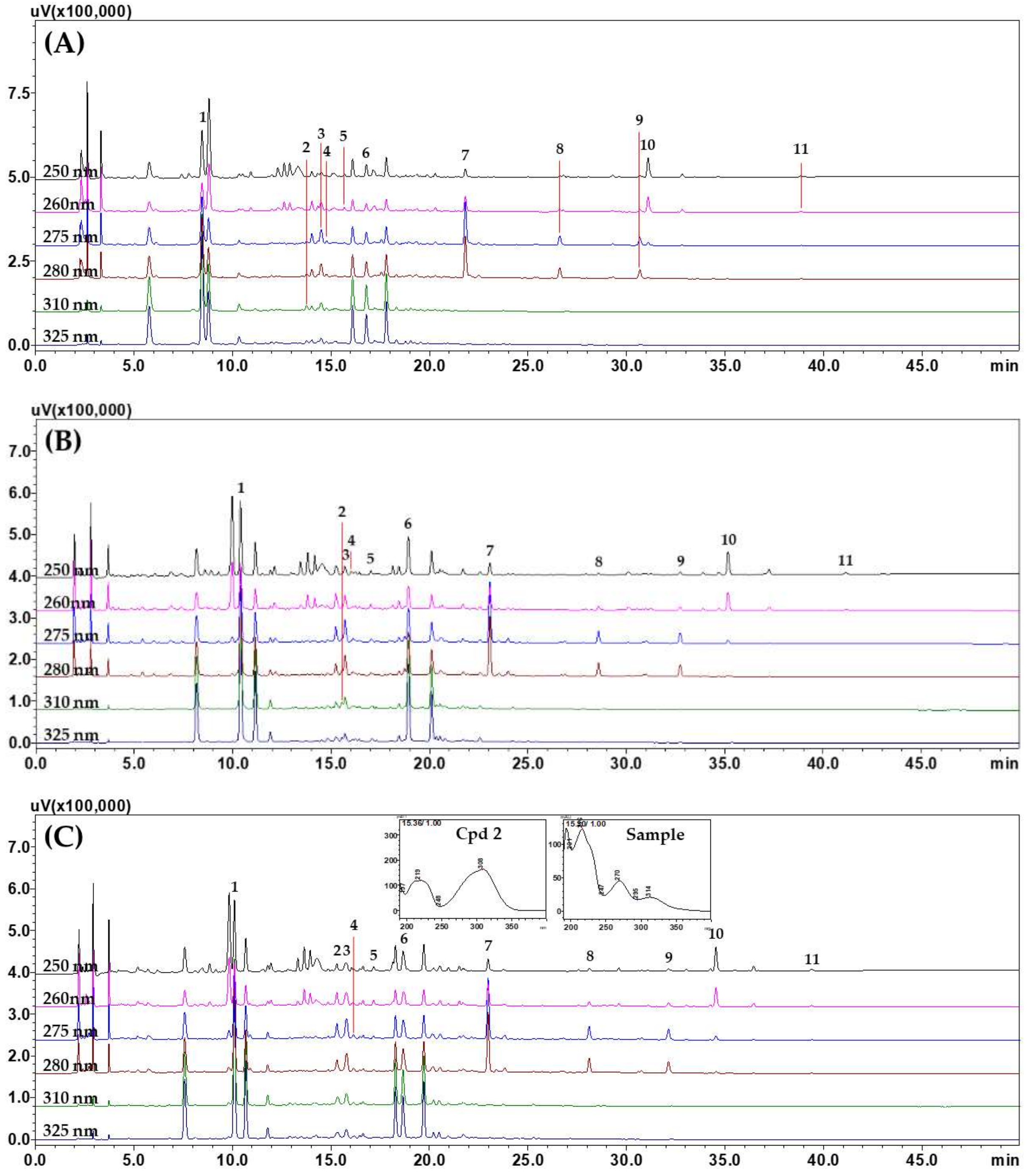

uV(x100,000)
(A)
(B)
(C)
250 nm
260nm
275 nm
280 nm
310 nm
325 nm
Cpd 2
Sample
min

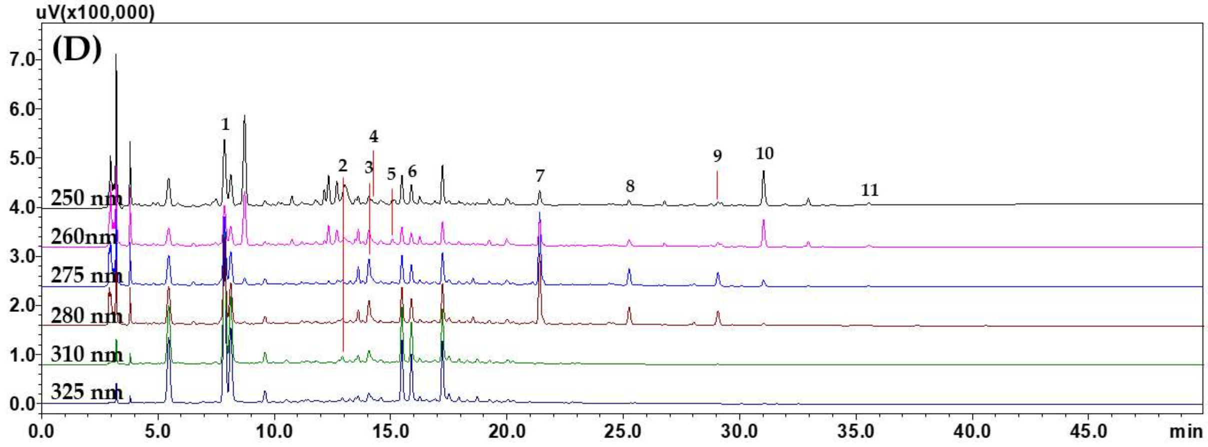

Figure S3. Comparison of HPLC chromatograms of marker compounds using different C_{18} columns. A: Capcell pak UG80, B: SunFire™, C: Gemini, and D: Hypersil GOLD. Chlorogenic acid (1), 4-hydroxycinnamic acid (2), liquiritin apioside (3), liquiritin (4), genistin (5), isochlorogenic acid A (6), arctiin (7), matairesinol (8), arctigenin (9), glycyrrhizin (10), and pulegone (11).

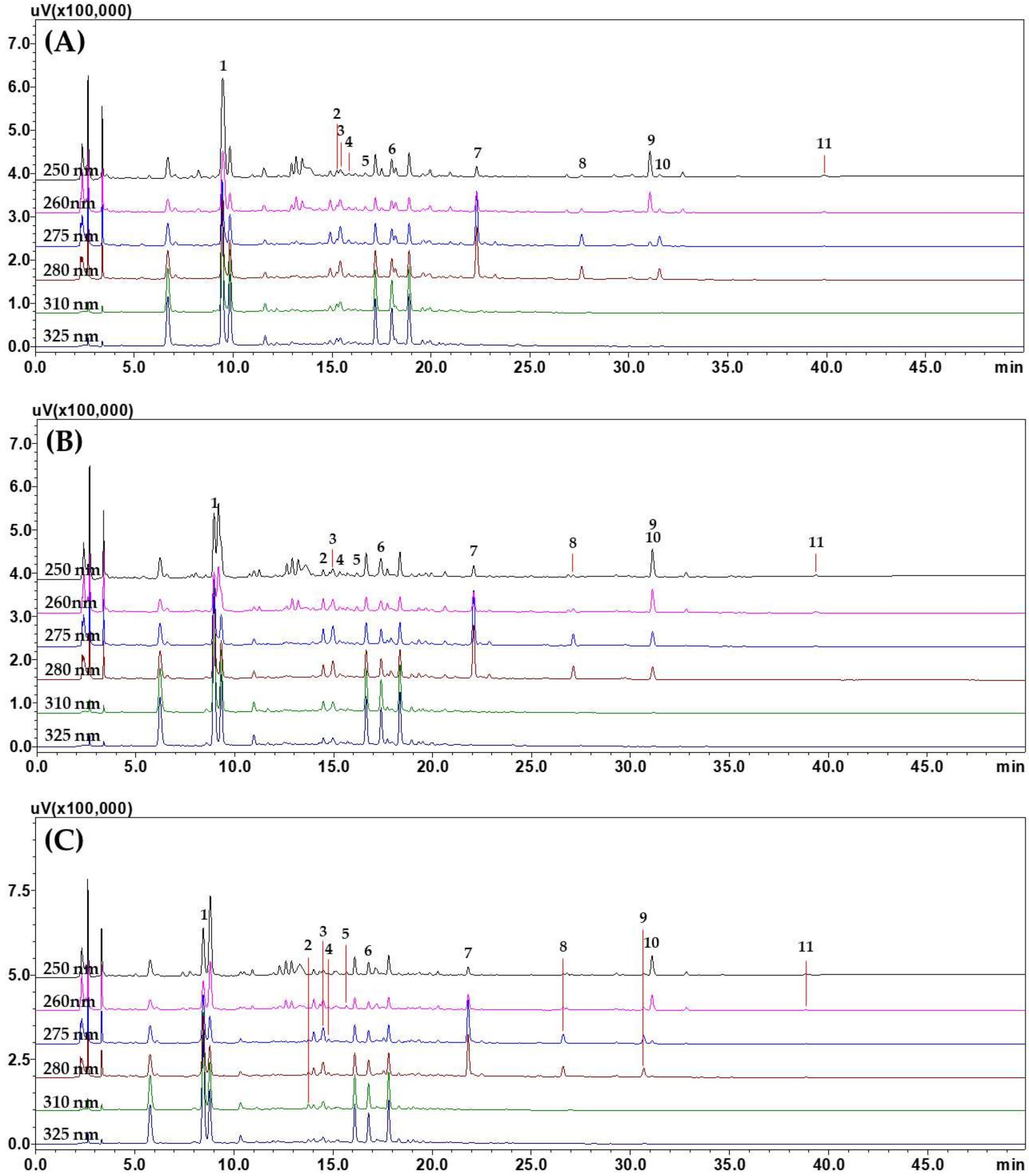

(A)
(B)
(C)
uV(x100,000)
250 nm
260nm
275 nm
280 nm
310 nm
325 nm
min

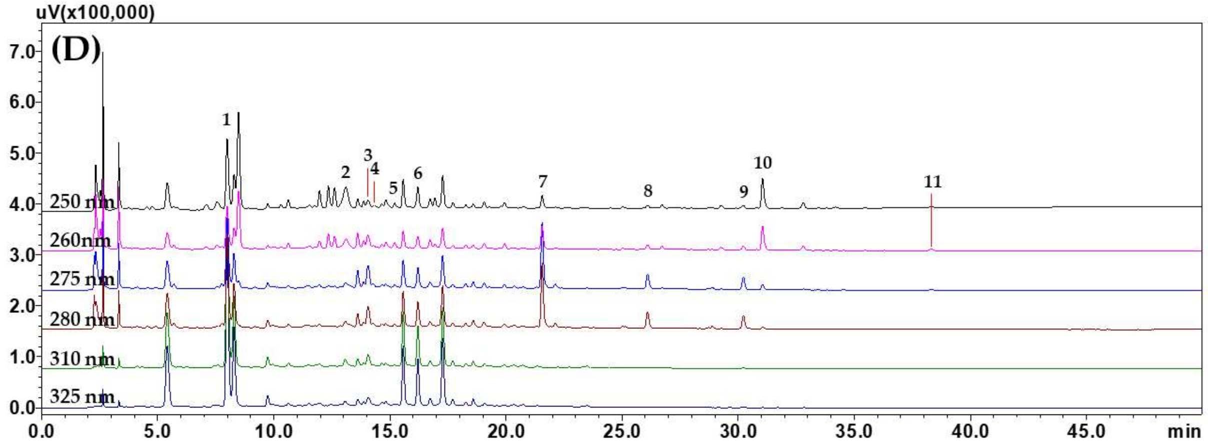

Figure S4. Comparison of HPLC chromatograms of marker compounds at different column oven temperatures. A: 30 °C, B: 35 °C, C: 40 °C and D: 45 °C. Chlorogenic acid (1), 4-hydroxycinnamic acid (2), liquiritin apioside (3), liquiritin (4), genistin (5), isochlorogenic acid A (6), arctiin (7), matairesinol (8), arctigenin (9), glycyrrhizin (10), and pulegone (11).

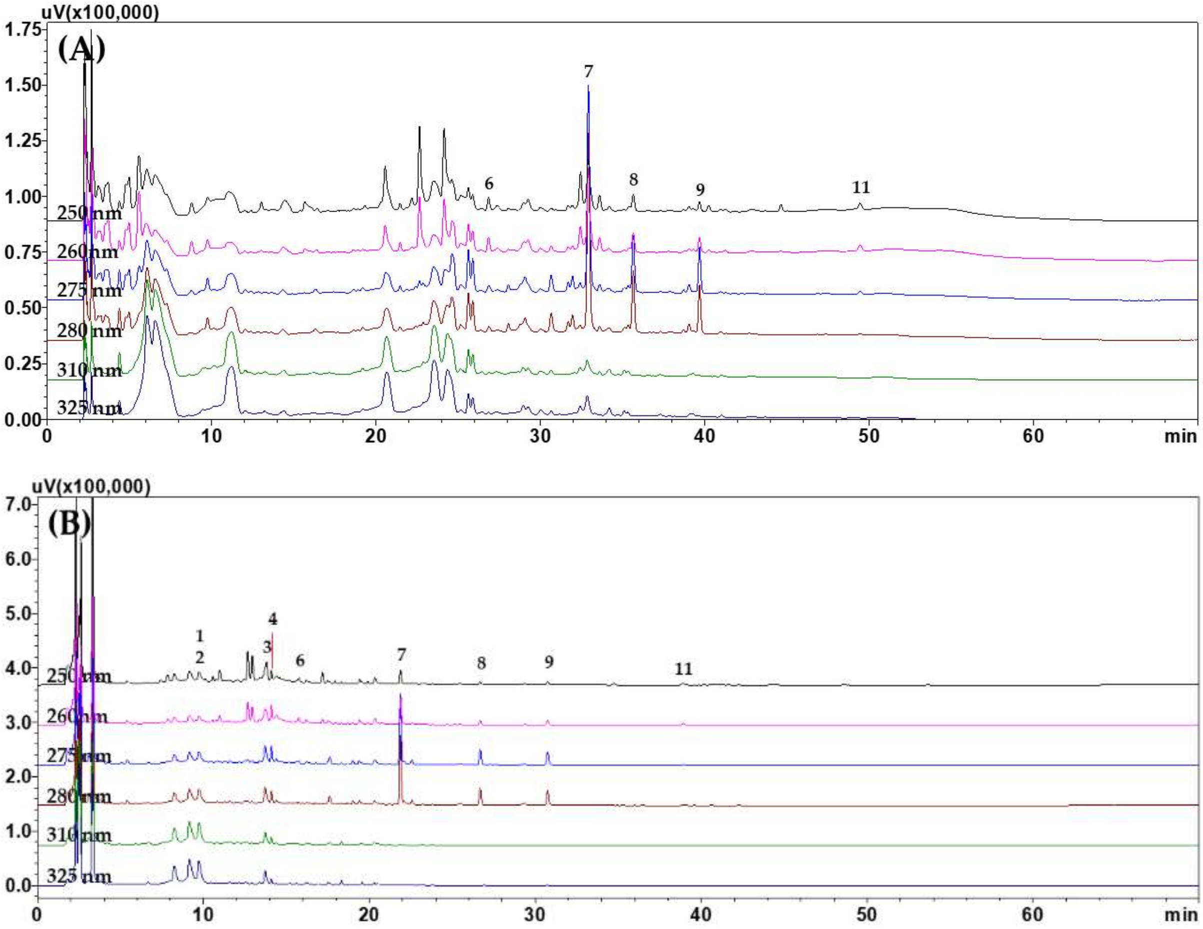

Figure S5. Comparison of HPLC chromatograms of marker compounds using different solvent systems. A: water–methanol system and B: water–acetonitrile system. Chlorogenic acid (1), 4-hydroxycinnamic acid (2), liquiritin apioside (3), liquiritin (4), genistin (5), isochlorogenic acid A (6), arctiin (7), matairesinol (8), arctigenin (9), glycyrrhizin (10), and pulegone (11).

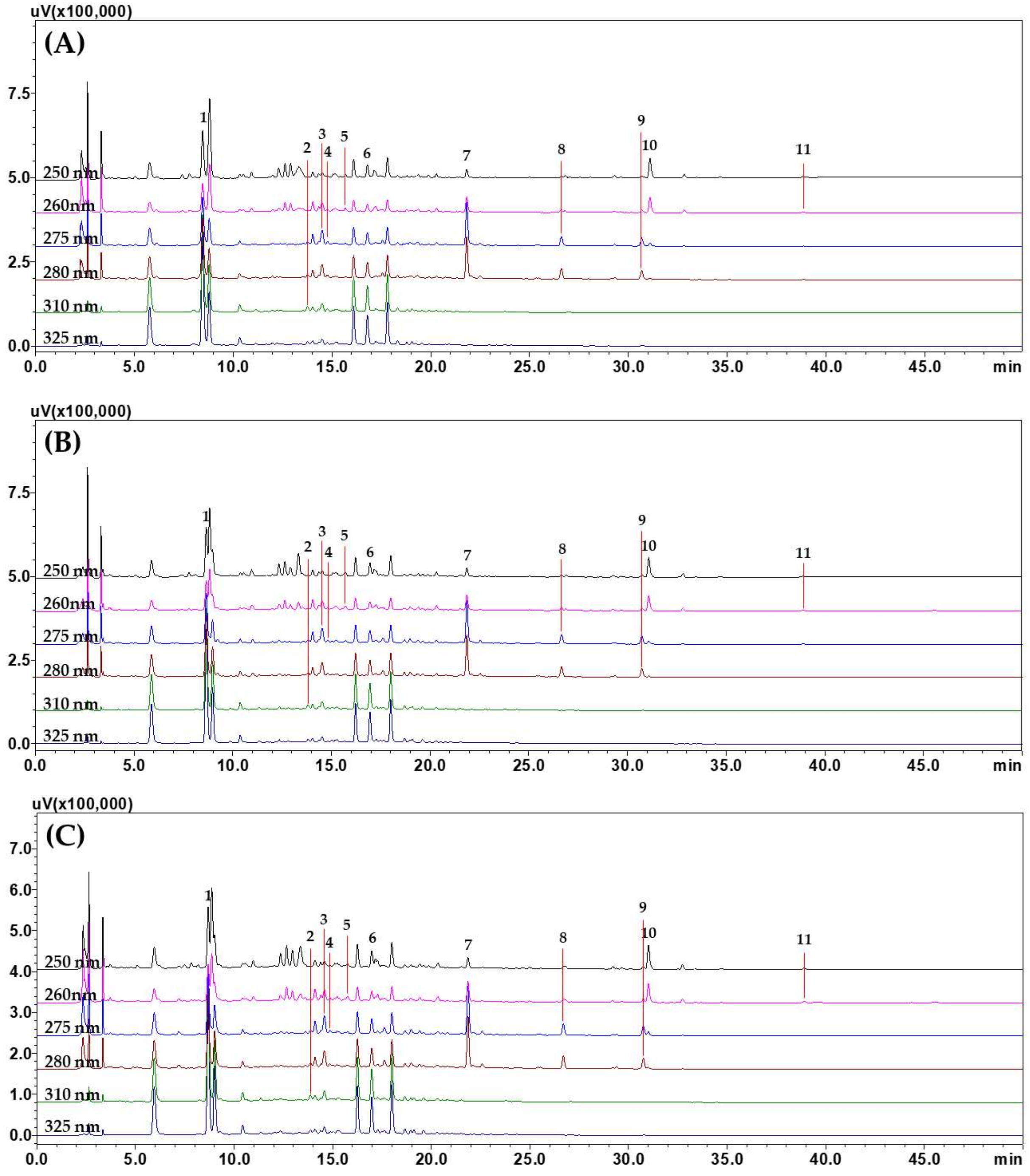
uV(x100,000)
(A)
(B)
(C)
250 nm
260nm
275 nm
280 nm
310 nm
325 nm
1
2
3
4
5
6
7
8
9
10
11
0.0
5.0
10.0
15.0
20.0
25.0
30.0
35.0
40.0
45.0
min

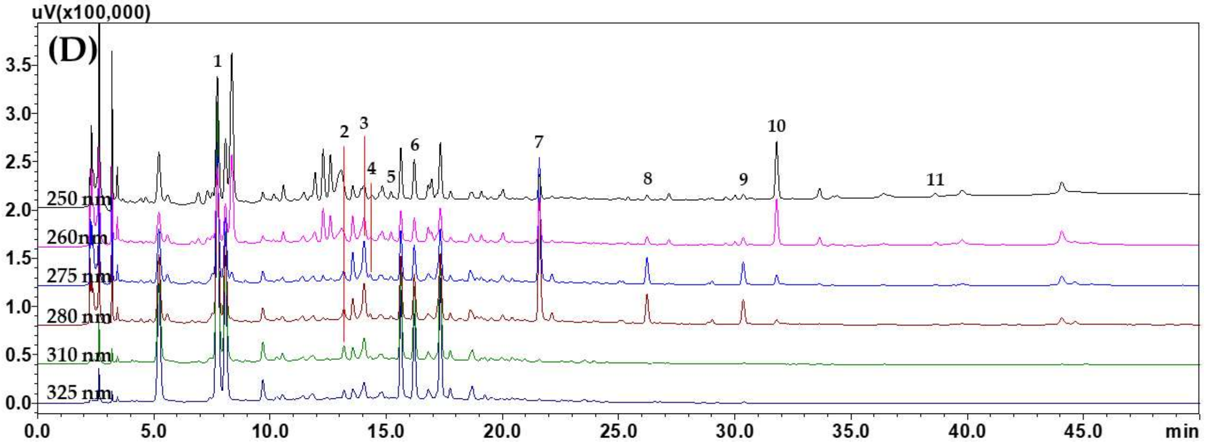

Figure S6. Comparison of HPLC chromatograms of marker compounds using different acidic modifiers in the mobile phase. A: 0.1% (v/v) formic acid, B: 0.1% (v/v) trifluoroacetic acid, C: 0.1% (v/v) phosphoric acid, and D: 1.0% (v/v) acetic acid. Chlorogenic acid (1), 4-hydroxycinnamic acid (2), liquiritin apioside (3), liquiritin (4), genistin (5), isochlorogenic acid A (6), arctiin (7), matairesinol (8), arctigenin (9), glycyrrhizin (10), and pulegone (11).

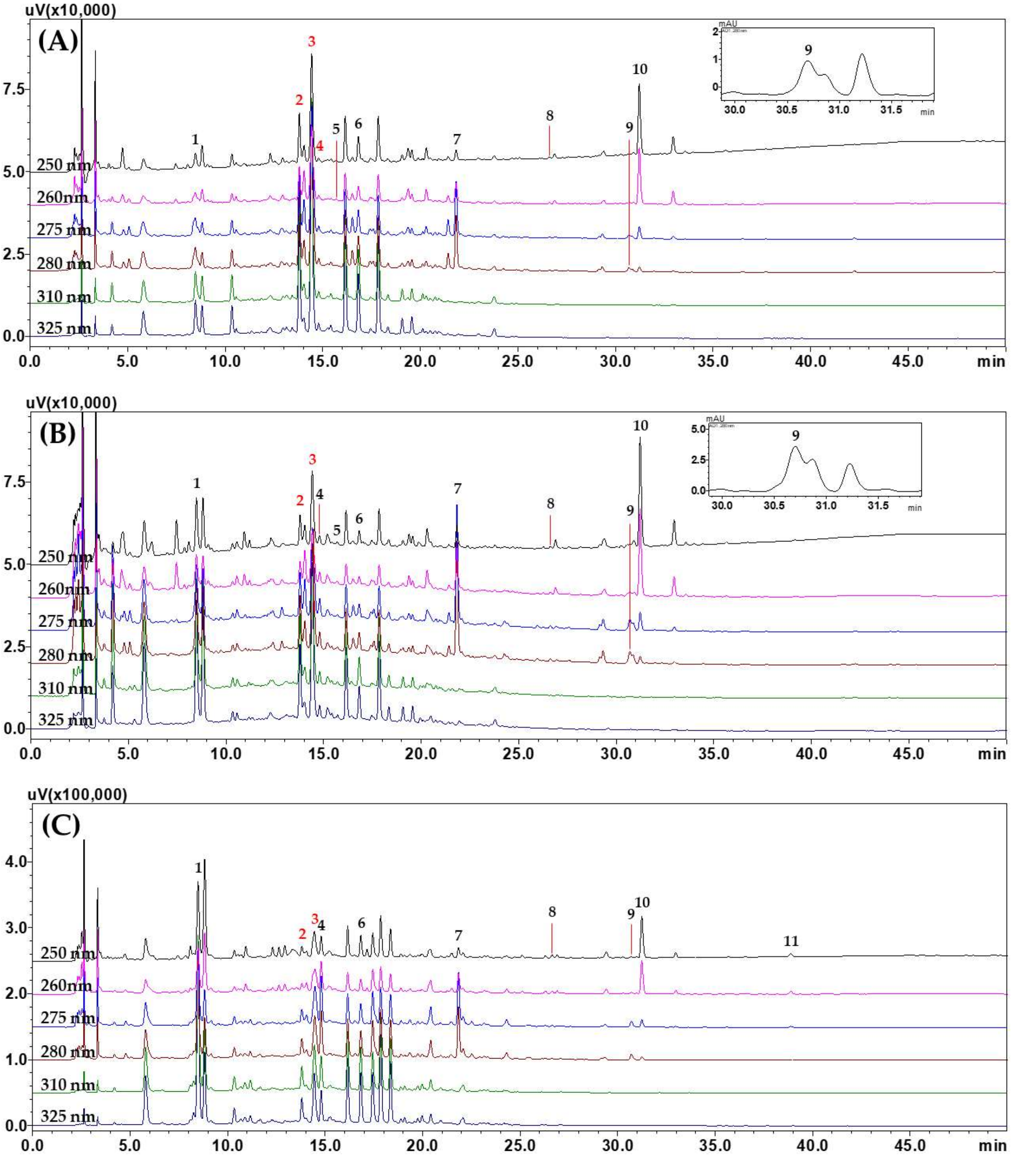

Figure S7. HPLC chromatograms of the commercial EGS formulations. A: EGS–2, B: EGS–3, and C: EGS–4. Chlorogenic acid (1), 4-hydroxycinnamic acid (2), liquiritin apioside (3), liquiritin (4), genistin (5), isochlorogenic acid A (6), arctiin (7), matairesinol (8), arctigenin (9), glycyrrhizin (10), and pulegone (11).

(A)

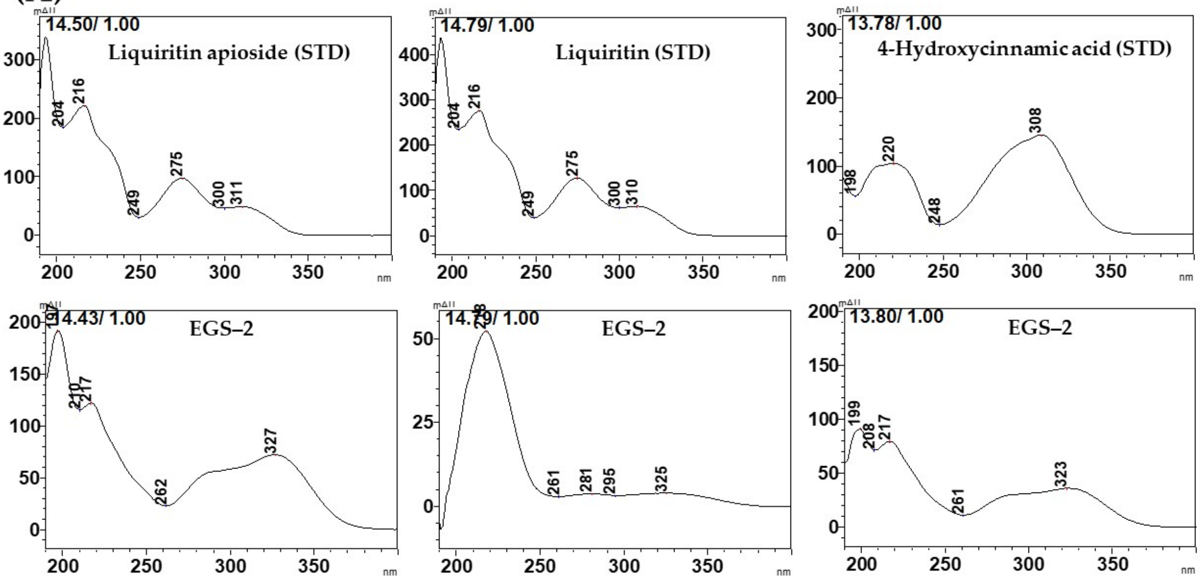

14.50/ 1.00
Liquiritin apioside (STD)
14.79/ 1.00
Liquiritin (STD)
13.78/ 1.00
4-Hydroxycinnamic acid (STD)
14.43/ 1.00
EGS–2
14.79/ 1.00
EGS–2
13.80/ 1.00
EGS–2
nm

(B)

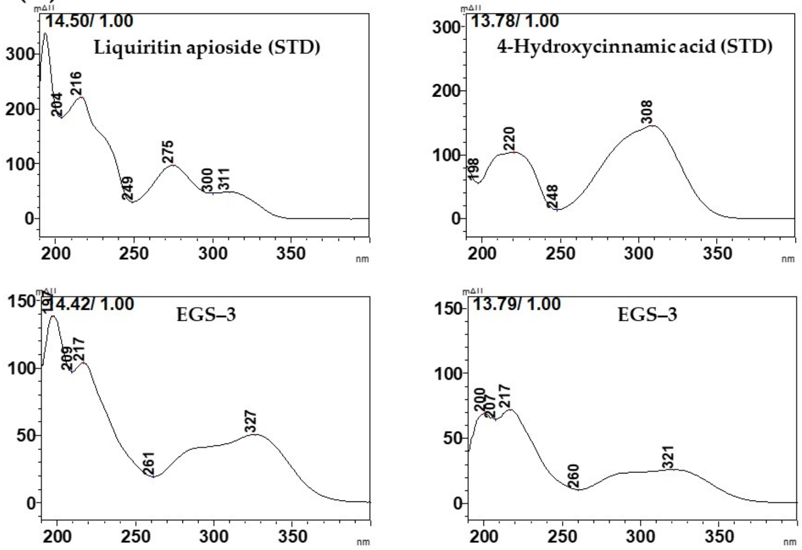

14.50/ 1.00
Liquiritin apioside (STD)
13.78/ 1.00
4-Hydroxycinnamic acid (STD)
14.42/ 1.00
EGS–3
13.79/ 1.00
EGS–3
nm

(C)

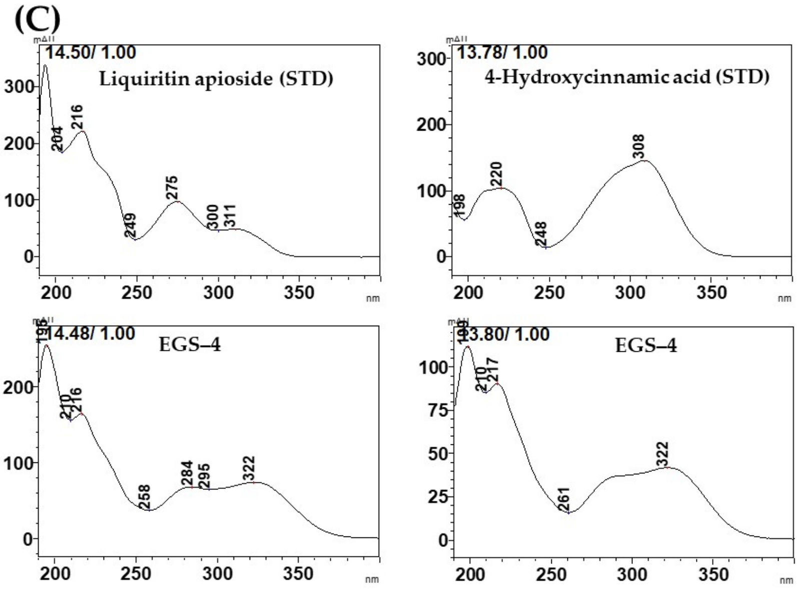

Figure S8. Comparison of UV spectral profiles between selected peaks in commercial EGS formulations and corresponding reference standards. A: EGS–2, B: EGS–3, and C: EGS–4.

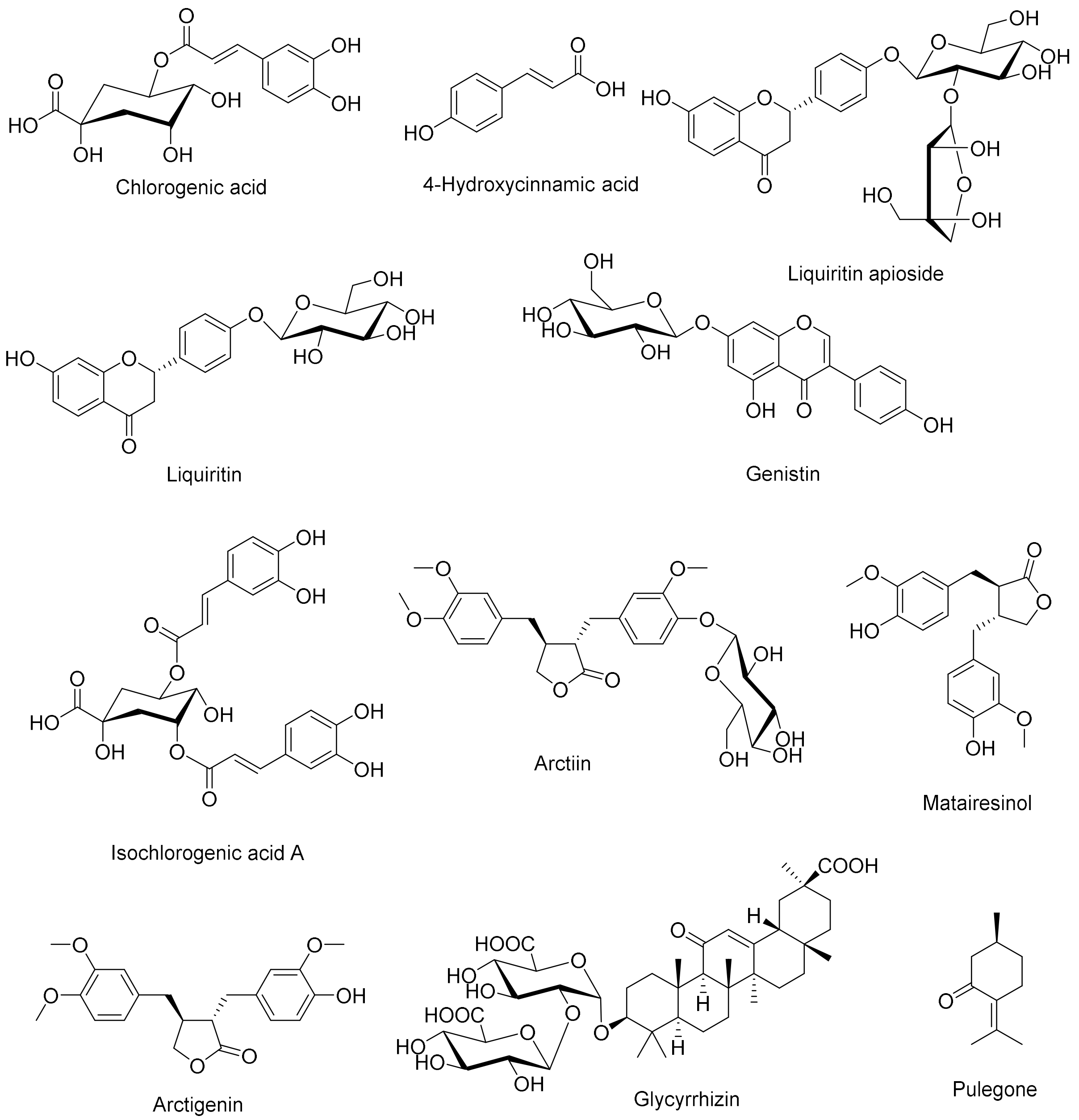

Figure S9. Chemical structures of 11 analytes selected as marker compounds for quality control of Eungyosan.
